# Deep Learning Parameter Estimation and Quantum Control of Single Molecules

**DOI:** 10.1021/acsphyschemau.6c00048

**Published:** 2026-06-12

**Authors:** Juan M. Scarpetta, Omar Calderón-Losada, Morten Hjorth-Jensen, John H. Reina

**Affiliations:** † Department of Physics and Centre for Bioinformatics and Photonics—CIBioFi, 28006 Universidad del Valle, Cali 760032, Colombia; ‡ Department of Physics and Center for Computing in Science Education, University of Oslo, Oslo N-0316, Norway

**Keywords:** single molecule, deep learning, ultrafast spectroscopy, quantum coherent control, open quantum systems

## Abstract

Coherent control, a central concept in physics and chemistry, has sparked significant interest due to its ability to fine-tune interference effects in atoms and individual molecules for applications ranging from light-harvesting complexes to molecular qubits. However, precise characterization of the system’s dissipative dynamics is required for its implementation, especially at high temperatures. In a quantum control experiment, this means learning system-bath parameters and driving coupling strengths. Here, we demonstrate how to infer key physical parameters of a single molecule driven by spectrally modulated pulses at room temperature. We develop and compare two computational approaches based on two-photon absorption photoluminescence signals: an optimization-based minimization scheme and a feed-forward neural network. The robustness of our approach highlights the importance of reliable parameter estimation in designing effective coherent control protocols. Our results have direct applications in ultrafast spectroscopy, quantum materials, and technology.

## Graphical abstract

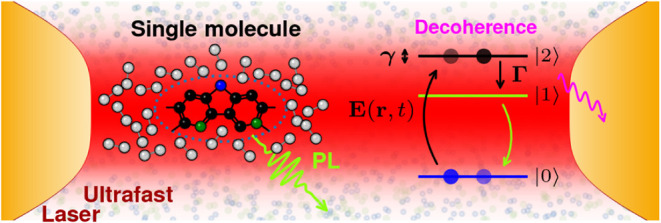

## Introduction

Coherent control is an experimental paradigm that has been extensively developed since the 1980s^1–3^ to study and manipulate the ultrafast dynamics of quantum systems using ultrashort laser pulses in the picosecond and femtosecond regimes.^4–8^ This strategy has also been implemented for developing molecular qubits in quantum information science.^9–12^ At these time scales, it is possible to probe and influence phenomena such as electronic dephasing,^13–15^ vibrational motion,^16,17^ stimulated emission,^18,19^ and charge/energy transfer processes.^20–24^ A wide variety of physical platforms, including semiconductor quantum dots,^25,26^ isolated atoms,^27^ molecular aggregates,^28^ and conjugated polymers,^22,29,30^ exhibit distinct transition energies and absorption cross-sections that enable the implementation of coherent control protocols. A central challenge here is the identification of optimal pulse sequences that facilitate targeted transitions between system levels while accounting for the diversity of spectral and dynamic properties across these platforms. In particular, multiphoton absorption processes^31,32^ are commonly exploited to create constructive or destructive interference between transition pathways,^33^ thereby enabling the coherent manipulation of the system dynamics.

For organic molecules and aggregates, one accessible observable is photoluminescence (PL). Several studies have measured PL from organic systems at room temperature, both at the single-molecule level and in ensembles.^34,35^ However, experimental constraints complicate these measurements, as many molecules undergo photobleaching on short time scales,^36^ and the emission yield must be sufficiently high for detection,^37^ which limits the achievable detection times. Moreover, environmental effects are particularly significant in ultrafast molecular experiments, as photophysical properties under ambient conditions deviate from those at low temperatures, resulting in substantially shorter dephasing times.^38^ Thus, single molecules must be considered open quantum systems that interact with the solvent and surrounding environment. Therefore, characterizing these dissipative effects, for example via spectral density functions^39–41^ or by estimating dephasing and relaxation rates,^41–47^ is essential, though not always feasible *a priori*. Despite this, many experiments have been successfully carried out on organic single molecules,^48,49^ dyes, and molecular samples with large two-photon absorption cross sections at room temperature.^50,51^ These experiments have measured two-photon PL emission using sequences of ultrashort laser pulses with spectral phase modulations^49,52^ as well as delayed pulse schemes.^51,53^

In this context, one molecule that has attracted much interest is the methyl-substituted ladder-type poly­(*para*-phenylene) (MeLPPP),^35,54^ a π-conjugated polymeric molecule with favorable photophysical properties.^55^ MeLPPP has been shown to be photochemically stable,^56^ exhibits a large two-photon absorption cross-section,^57^ and can be detected at the single-molecule level. These properties make MeLPPP a strong candidate for room-temperature coherent control experiments.^52,53^ However, as with all complex molecular systems, obtaining reliable information about the environment and the system–field coupling from PL measurements requires careful modeling of dissipative processes and an understanding of the relevant energy and temporal scales.

To address these challenges, research on quantum noise spectroscopy and open quantum dynamics has focused on automating the extraction of relevant environmental features and parameters in a robust, reliable, and interpretable manner.^58,59^ Machine learning and deep learning techniques have been proposed and applied to infer physical parameters from dynamical observables,^45,60–62^ for instance, in two-level systems^45,63^ and related models.^64,65^ These approaches aim to recover dissipative and driving parameters from experimentally accessible signals while minimizing the required prior knowledge about the system–environment coupling.^66,67^

An algorithm for PL measurements of the MeLPPP molecule, termed quantum dynamics identification (QDI), has been reported in ref 52. QDI minimizes the residuals between the experimental data and the PL model and has been developed to estimate parameters associated with dissipative effects and system–environment coupling. However, algorithms of this type are sensitive to the chosen initial conditions and must be rigorously tested to determine their reliability and convergence behavior.

In this work, we present two computational approaches for extracting key physical parameters, including dephasing and relaxation rates, system–field coupling, and energy levels, from a single π-conjugated MeLPPP molecule^68,69^ driven by spectrally modulated pulses with phase and amplitude shaping. We calculate the ultrafast dissipative dynamics and characterize how the control parameters influence the photoluminescence signal generated via two-photon absorption. The first method is an optimization-based scheme that minimizes a defined loss function for PL, while the second method uses a feed-forward neural network trained to map PL traces onto the target parameters. We assess the robustness of both approaches under different preprocessing strategies and quantify the associated uncertainties in the inferred parameters. The experimental PL data used for final validation correspond to single-molecule MeLPPP measurements.^49,52,53^

## Methods

### Theoretical Framework

The molecular system can be described by a three-level time-dependent Hamiltonian of the form

1 $$Ĥ\left(t\right)={Ĥ}_{0}+f\left(t\right){Ĥ}_{1}$$

where *Ĥ*_0_ is diagonal with respect to its electronic energies *E*_0_, *E*_1_, and *E*_2_. The function *f*(*t*) represents the time-dependent driving corresponding to the phase-modulated pulses, and *Ĥ*_1_ accounts for the coupling between the states associated with the transitions. Specifically, the Hamiltonian of a nonlinear two-photon absorption process between the energies *E*_0_ and *E*_2_ ^70–72^ is represented as

2 Ĥ(t)=[E00−Ω2PRe{E2(t)}0E10−Ω2PRe{E2(t)}0E2]

where $\Omega_{2P}\equiv \mu_{02}^{2}E_{0}^{2}$ denotes the two-photon Rabi frequency, *E*_0_ is the peak strength of the unchirped laser field, and μ_02_ is the effective dipolar moment of the *S*_0_ → *S*_2_ transition (see Figure 1a). *E*(*t*) represents the normalized (complex) electric field of the chirped laser pulse. In the frequency domain, this electric field is expressed as *E*(ω) = *A*(ω)­e^i*φ(ω)*^, comprising both spectral amplitude and phase components. In the time domain, the intensity field reads

3 $$E\left(t\right)=\frac{1}{\sqrt{2\pi }}\int_{-\infty }^{\infty }A\left(\omega \right)M\left(\omega ,\tau \right)e^{i\varphi (\omega ,\tau ,\beta )}e^{-i\omega t}d\omega$$

where *A*(ω) represents the laser spectrum function (see, e.g., Figure 2a). The parameters τ and β control pulse shaping and comprise both spectral phase modulation φ­(ω, β, τ), with quadratic chirp β, and amplitude mask modulation *M*(ω, τ), with delay time τ. These modulations affect the driving effects on the system dynamics and control the photoluminescence of the molecule.^70^ The modulated field profiles are shown in Figure 2, where panel (a) shows the pulse spectrum, while panels (b) and (c) depict the corresponding pulse broadening and splitting induced by the control parameters.

**Figure 1:**
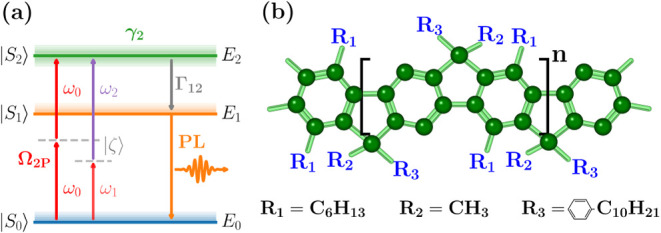
(a) Schematics of the individual molecules’ energy levels description. *E_i_* denotes the three-level energies of the molecule with corresponding two-photon absorption transition between *E*_0_ and *E*_2_ at Rabi frequency Ω_2P_ and |*S_i_* ⟩ ≡ |*i*⟩ represents the electronic states. The two-photon transitions occur via the virtual states |ζ⟩. The relaxation processes between levels *E*_2_ → *E*_1_ occur at a rate Γ_12_, and dephasing in the singlet state *S*_2_ at a rate γ_2_. (b) Molecular structure of MeLPPP (R1, *n*-hexyl; R2, methyl; R3, 1,4-decylphenyl).

**Figure 2:**
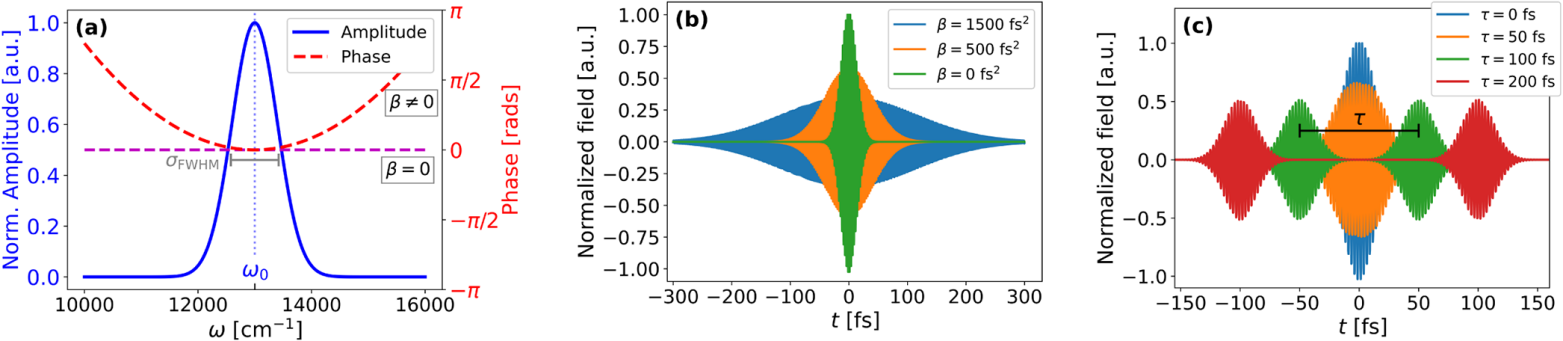
(a) Frequency domain profile of the field. The solid line represents the normalized spectrum *A*(ω) with central frequency ω_0_ = 12 987 cm^–1^ and spectral width σ_fwhm_ = 400 cm^–1^. The dashed lines represent the spectral phase φ­(ω) for chirped (β ≠ 0) and unchirped (β = 0) fields. (b) Normalized temporal profile of the chirped field (see eq 5) for different β values. (c) Normalized temporal profile of the field with amplitude and phase modulation masks (see eq 7) for different τ values.

The dynamics of the electronic populations and coherences of an *N*-level system embedded in a dissipative environment and driven by the time-dependent Hamiltonian eq 2 can be described by the density operator *ρ̂*, as follows:^47^

4 $$\frac{d}{dt}\mathrm{\rho ̂}\left(t\right)=\frac{-i}{\hbar }\left[Ĥ\left(t\right),\mathrm{\rho ̂}\right]+\overset{N-1}{\underset{i\neq j=0}{\sum }}L_{ij}\left(\mathrm{\rho ̂}\right)+\overset{N-1}{\underset{i=0}{\sum }}D_{i}\left(\mathrm{\rho ̂}\right)$$

with superoperators $L_{ij}=\Gamma_{ij}\left(\rho_{ii}\left|j\right\rangle \langle j|-\frac{1}{2}\left\{\left|i\right\rangle \langle i|,\mathrm{\rho ̂}\right\}\right)$ and $D_{i}=\frac{\gamma_{i}}{2}\left(\rho_{ii}\left|i\right\rangle \langle i|-\frac{1}{2}\left\{\left|i\right\rangle \langle i|,\mathrm{\rho ̂}\right\}\right)$ . Here, ρ_*ij*_, with *i*, *j* = 0, 1, 2, denotes the matrix elements of the density operator, and the computational basis {|0⟩, |1⟩, |2⟩} is used. $L_{ij}$ and $D_{i}$ describe the relaxation processes between two energy levels *E*_*j*_ → *E*_*i*_ at a rate Γ_*ij*_ and dephasing in the state *S*_*k*_ at a rate γ_*k*_, respectively. Within this formalism, γ_*i*_ and Γ_*ij*_ completely encode the system–environment quantum dissipative effects.

### Numerical Simulations

For the three-level system depicted in Figure 1a, we consider the initial ground state *ρ̂*(0) = |0⟩⟨0|. Over time, the system is driven by the pulse *E*(*t*), resulting in a transient population transfer between states *S*_0_ and *S*_2_ via a two-photon absorption process. The population in the virtual state *S*_2_ then undergoes dephasing at a rate γ_2_ and eventually relaxes to the *S*_1_ level at a rate Γ_12_. At later times, compared to the pulse duration, the populations of the three levels decay to their steady-state values, with the photoluminescent state *S*_1_ becoming predominant.

For the MeLPPP molecule, the eigenenergies *E*_0_ = 0 and *E*_1_ = 22 000 cm^–1^ are retrieved from the one-photon emission characteristic spectrum.^35,54^ The *S*_2_ energy *E*_2_ = 25 940 cm^–1^, relaxation rate Γ_12_ = 1/190 fs^–1^, and dephasing rate γ_2_ = 1/61 fs^–1^ are taken from the numerical results reported in refs 52 and 53. For red-detuned light from a typical Titanium Sapphire laser, the Gaussian spectrum is centered at λ_0_ = 770 nm (12 987 cm^–1^) with a spectral full width at half-maximum (fwhm) σ_fwhm_ = 24.3 nm, corresponding to a Fourier-limited pulse of 40 fs.

In a first approach, we consider a modulation applied only to the spectral phase in quadratic form

5 $$M\left(\omega ,0\right)=1,,\varphi \left(\omega ,\beta \right)=\frac{1}{2}\beta {\left(\omega -\omega_{0}\right)}^{2}$$

with no modulation in the spectral amplitude. Figure 3 shows the population evolution of the MeLPPP system for different values of β, demonstrating that the control parameter clearly reshapes the coupling and transient effects of the levels.

**Figure 3:**
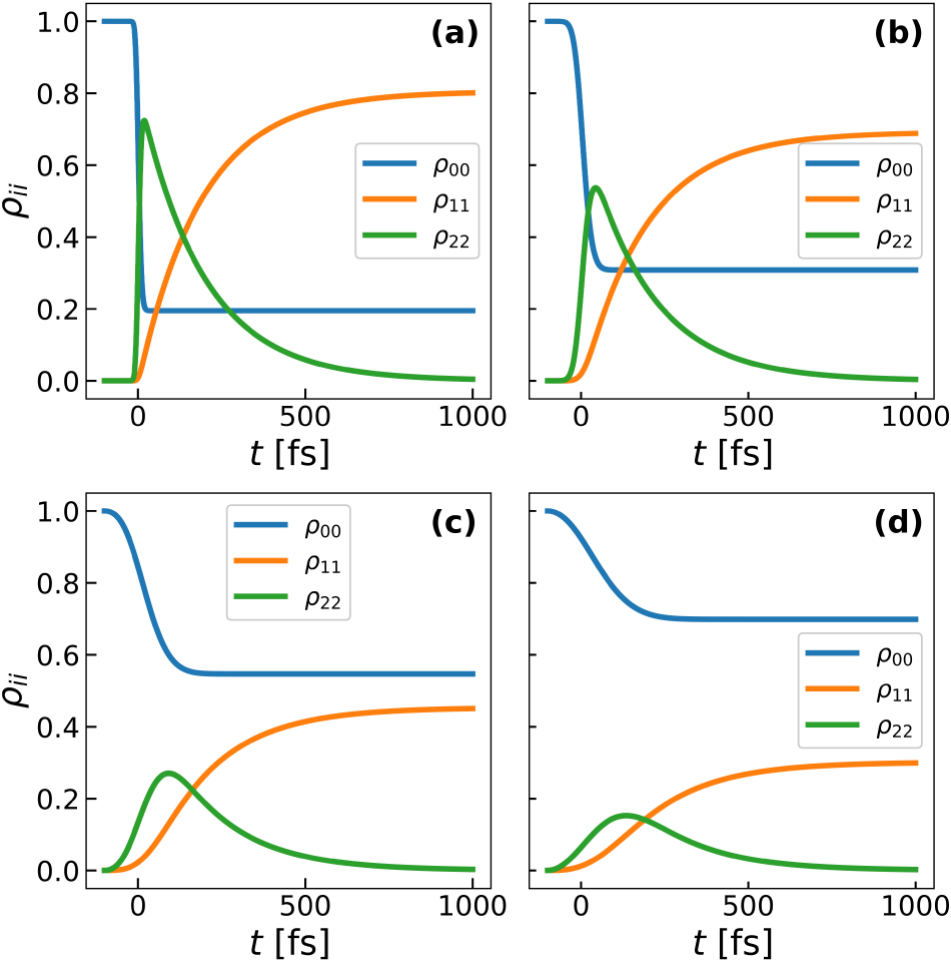
Population dynamics of the MeLPPP molecule for Ω_2P_ = 531 cm^–1^, γ_2_ = 1/61 fs^–1^, Γ_12_ = 1/190 fs^–1^, and control parameter values: (a) β = 0 fs^2^, (b) β = 500 fs^2^, (c) β = 1500 fs^2^, and (d) β = 2500 fs^2^.

At long times, the populations reach their steady values, allowing the evaluation of the expected PL associated with the corresponding quantum dynamics. This is shown in Figure 3. For small values of β (β < 1000 fs^2^), the *S*_1_ state is predominantly reached. For larger values, however, there is less excitation of *S*_1_, resulting in a lower PL signal. Since the pulse *E*(*t*) is centered at *t* = 0 fs, the simulations are initialized at *t* = −100 fs to ensure that the dynamics start prior to the peak of the laser field. While this choice may place some chirped pulses partially within the simulation window (see Figure 2b and c), it does not affect the long-time behavior. The steady-state values are evaluated at *t* = 1 ps.

As a second approach, we consider spectral and phase modulation as follows:

6 $$M\left(\omega ,\tau \right)=\left|\cos\left(\frac{1}{2}\left(\omega -\omega_{0}\right)\tau \right)\right|$$

7 $$\varphi \left(\omega ,\tau \right)=\frac{\pi }{2}\mathrm{sgn}\left[\cos\left(\frac{1}{2}\left(\omega -\omega_{0}\right)\tau \right)\right]$$

where sgn(·) represents the sign function. Figure 2 shows the modulation effects on the pulses. The results for populations driven by these pulses are shown in Figure 4.

**Figure 4:**
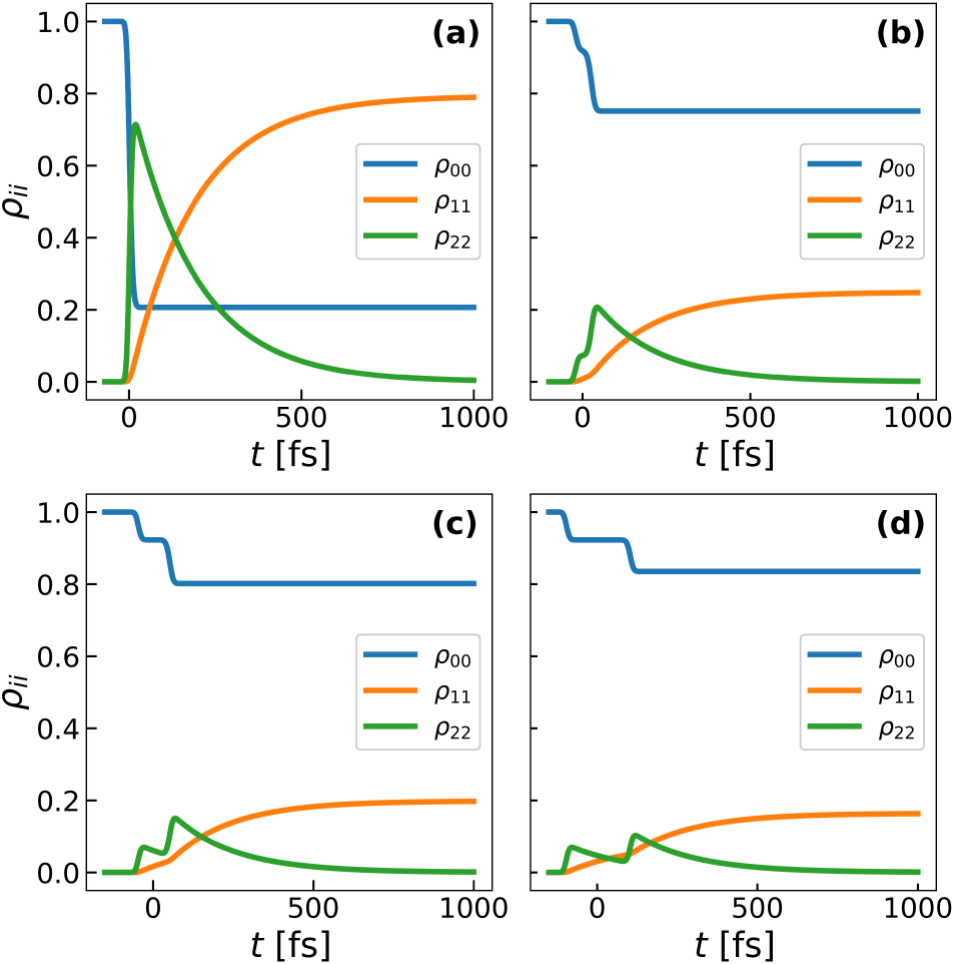
Ultrafast population dynamics of the MeLPPP molecule with values Ω_2P_ = 531 cm^–1^, γ_2_ = 1/61 fs^–1^, Γ_12_ = 1/190 fs^–1^, and different control parameters: (a) τ = 0 fs, (b) τ = 50 fs, (c) τ = 100 fs, and (d) τ = 200 fs.

For this second modulation, the population ρ_11_ reaches its maximum at small values of τ (τ < 50 fs), corresponding to pulses that are split into two subpulses of half amplitude (see Figure 2c). Consequently, this modulation offers only a limited degree of control over the PL intensity.

As can be seen in Figures 3 and 4, the control parameters β and τ decisively determine the long-time behavior of the system. These parameters govern the final population of the steady state, particularly that of state ρ_11_, the target level accessed via the two-photon absorption process. The behavior of ρ_11_ provides direct information about the photoluminescence of the molecular sample because the PL intensity is linearly related to this density matrix element.^52,53^

Figure 5 shows the complete time evolution of ρ_11_ as a function of the two control parameters, illustrating how the system’s ultrafast dynamics and steady-state behavior are shaped by the coherent control conditions. This representation highlights the strong sensitivity of ρ_11_ to phase dispersion and temporal delay parameters, providing direct insight into the interplay between field coherence, phase modulation, and the molecular response.

**Figure 5:**
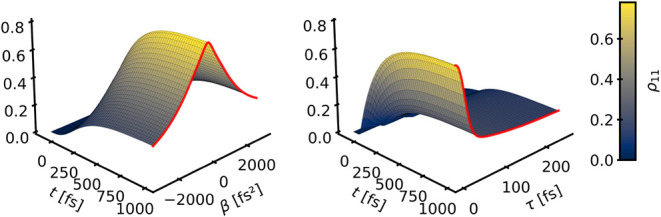
Full map of the ultrafast dynamics of the population ρ_11_ of the MeLPPP molecule as a function of time and the control parameters β and τ. At steady-state times (∼1 ps), the obtained values reproduce the shape of the PL signal, as indicated by the red solid line. Maximum values of ρ_11_ occur at β = 0 and τ = 0, while deviations from these conditions lead to distinct PL intensities.

These simulations reveal that the parameter τ leads to a pronounced maximum in the PL signal; however, the response rapidly decays as τ deviates from its optimal value. In contrast, the chirp parameter β produces symmetric PL intensities around β = 0 and enables smoother, more controlled exploration of the parameter space. For this reason, the remainder of this work focuses on algorithms and protocols that rely on PL measurements as a function of the chirp parameter.

### Optimization Methods

In this section, we present two different numerical approaches for extracting relevant dissipative bath and driving coupling parameters from PL data. The first approach is based on numerically optimizing nondifferentiable functions using standard simplex methods, such as the Nelder–Mead algorithm implemented in SciPy.^73^ The second approach employs a feed-forward neural network that takes the PL data as input.

#### Parameter Selection and Constructed Data Sets

The dynamics and final populations are sensitive to specific parameters related to environmental dissipation and field coupling strength, which lead to appreciable effects on the stationary final state and, consequently, on the experimentally measured photoluminescence over the ensemble of molecules. As a result of collective emission, the steady-state value of the single-molecule population ρ_11_ is related to the photoluminescence function PL(·) as^52,53^

8 $$\mathrm{PL}\left(Q\right)=A\rho_{11}\left(Q\right)+B$$

where the constants *A* and *B* scale the experimentally measured PL signal to match the total emission intensity from all molecules within the laser spot and the dark-count background noise, respectively. Since no a priori information is available about the bath effects driving the dissipative dynamics or about the optimal field–system coupling, we selected the parameter set $Q=\left[E_{2}\Omega_{2P}\gamma_{2}\Gamma_{12}\right]$ as free variables to analyze the dependence of the ultrafast dynamics revealed in the PL signal. The remaining parameters were kept fixed, as they correspond to laser configurations and molecular properties that are not optically addressed by the excitation pulses.^35,53^

To evaluate both algorithms, the experimental PL data reported in ref 52 were used, where single-molecule MeLPPP dissolved in toluene (Sigma-Aldrich, 99.7%) at a concentration of 10^–9^ M, containing 5 mg/mL polystyrene, was employed. This measured PL signal ranges from 169 to 705 cps using 55 different β values. Therefore, we set the scaling parameters to *A* = 742.88 cps and *B* = 43.73 cps.

Accordingly, for the first optimization algorithm, a data set D of 700 parameter sets was generated to represent the initial conditions of the parameters Q by randomly sweeping the values within their physical ranges. Two scenarios were considered. The first one includes the full parameter set $Q=\left[E_{2}\Omega_{2P}\gamma_{2}\Gamma_{12}\right]$ , incorporating both the energy *E*_2_ of the virtual state *S*_2_ reached after two-photon excitation and the remaining parameters associated with driving and environmental decay processes. In the second scenario, the approximation *E*_2_ = 2ω_0_ was applied, assuming that the two absorbed photons have an energy equal to the spectral peak, since the spectral fwhm is small compared with the central wavelength.^74^ Under this approximation, the optimization problem is reduced to three free parameters. In both cases, these parameters were scanned in the same way. The two-photon Rabi frequency was swept over the range [200 cm^–1^, 800 cm^–1^], while the dephasing rate values were varied within [0.005 fs^–1^, 0.05 fs^–1^] and the decay rate values within [0.002 fs^–1^, 0.01 fs^–1^]. These values are consistent with the ultrafast femtosecond time scale of dephasing and decay processes,^52,56,75^ corresponding to dephasing times from 20 fs up to 200 fs and relaxation times from 100 fs up to 500 fs. For the first scenario, *E*_2_ was varied within the range [22 000 cm^–1^, 30 000 cm^–1^], whereas in the second scenario, the energy was fixed at *E*_2_ = 2ω_0_ = 25 940 cm^–1^.

For the NN-based approach, a PL data set was constructed consisting of *N* = 10 000 training samples, each containing 55 features corresponding to intensity PL values for different β values uniformly distributed within the range [−3000 fs^2^, 3000 fs^2^]. This data set was generated by fixing *E*_2_ = 2ω_0_ and sweeping the parameters $Q=\left[\Omega_{2P}\gamma_{2}\Gamma_{12}\right]$ within the same intervals as before, and then computing the PL using eq 8. These parameters Q also represent the target quantities to be predicted and evaluated on the testing data set.

Since the PL inputs and target variables span heterogeneous scales, ranging from tens to thousands of cm^–1^ for the energy and Rabi frequency, and from 10^–2^ to 10^–3^ fs^–1^ for the decay rates, three scalers were considered for normalization and rescaling of the parameters: Standard Scaler, Robust Scaler, and Robust Scaler with a Winsorization Preprocessing (WP).^76,77^ These feature scaling techniques help to uniformly distribute the input and target values, facilitating the NN training by keeping the outputs on the order of unity, which matches the typical magnitude of their weights, biases, and activation functions. Furthermore, the standard algorithms for numerical optimization also require consistent parameter scales to ensure proper convergence and computational efficiency.^78^

#### Optimization Algorithm

Using the set of experimental observations defined as {(β, *y*_β_): β = β_1_, β_2_,···,β_*n*_}, where *y*_β_ denotes the measured PL intensity corresponding to a specific value of the control parameter β, we define the mean squared error (MSE) metric as

9 $$\mathrm{MSE}=\frac{1}{n}\underset{\beta }{\sum }{[y_{\beta }-{\mathrm{PL}}_{\beta }(Q)]}^{2}$$

where *n* represents the total number of experimental data points. In our case, we selected the experimental outcomes reported in ref 52 as targets for parameter prediction. Additionally, we impose the constraint that the energy of the virtual state *S*_2_, denoted by *E*_2_, lies within an energy band close to 2ω_0_, which corresponds to approximately twice the energy of the laser spectral peak, as expected in a two-photon absorption process. To account for this physical constraint, we introduce a penalization term to the MSE, yielding the total loss function

10 $$L\left(Q\right)=\frac{1}{N}\underset{\beta }{\sum }{[y_{\beta }-{\mathrm{PL}}_{\beta }(Q)]}^{2}+\lambda {\left(E_{2}-2\omega_{0}\right)}^{2}$$

where λ is the hyperparameter that controls the strength of the constraint. The case λ = 0 corresponds to the first scenario, in which the approximation *E*_2_ = 2ω_0_ is imposed, and the optimization is reduced to three free parameters. Conversely, the case λ ≪ 1 represents the full optimization problem, where *E*_2_ is treated as an additional free variable.

By minimizing the loss function in eq 10, we obtain the optimal set of parameters that best relate the experimental outcomes to the numerical simulations. Hence, the molecular and bath properties can be extracted from these measurements by solving the optimization task

11 $$Q^{*}=\arg\underset{D}{\min}L\left(Q\right)$$

where $Q^{*}$ denotes the optimal parameter configuration. We employ the standard minimize function from SciPy using the Nelder–Mead algorithm^73^ to minimize the loss function in eq 10 across the parameter set Q . This choice is motivated by the fact that the problem involves the minimization of a black-box function with no analytical gradients and computationally expensive evaluations. A total of 700 initial conditions from D were tested in parallel to ensure convergence robustness. The optimization was carried out with a maximum of 500 iterations and a function tolerance (ftol) of 10^–4^, which provided a good balance between accuracy and computational efficiency.

#### Feed-Forward Neural Network

As an alternative approach, we implemented a neural network model that takes the PL values as input features and returns predicted physical parameters. In this framework, we aim to find an optimal mapping function represented by a feed-forward neural network (FFNN) that learns the relationships and underlying patterns between the PL inputs and their associated parameters Q , as illustrated in Figure 6. To obtain a highly accurate and sensitive predictor, we performed a systematic exploration of different neural network architectures and training strategies to evaluate their performance on unseen data.

**Figure 6:**
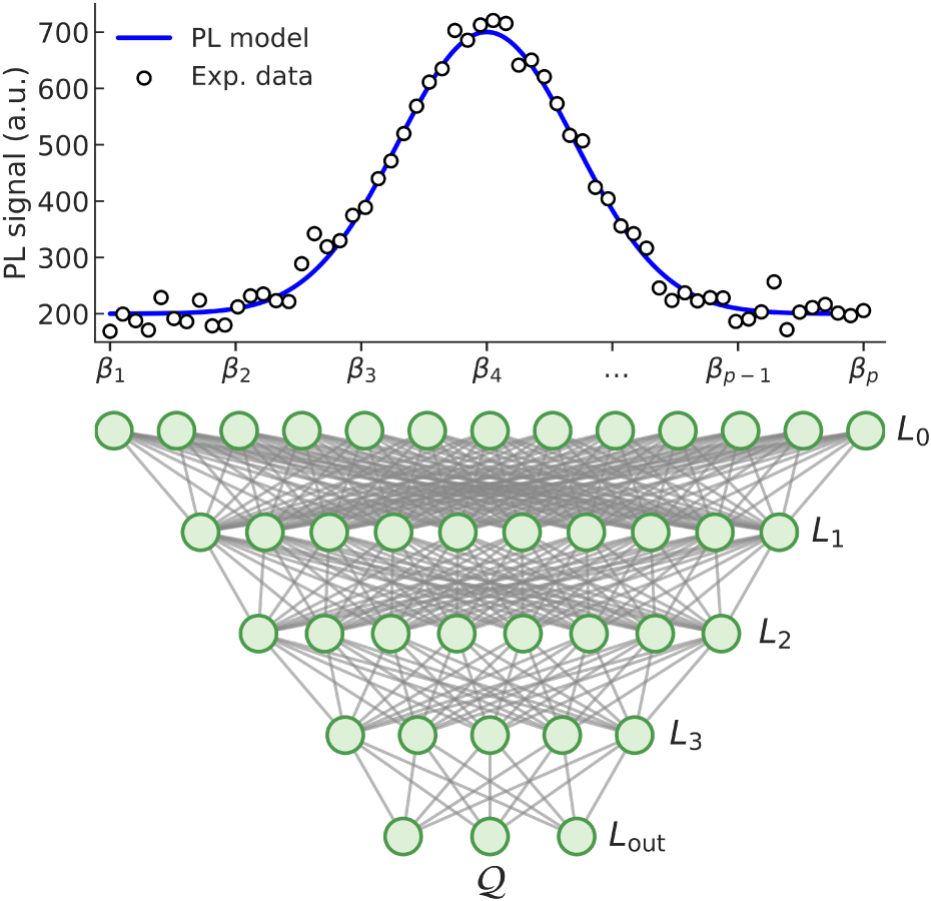
Sketch of the feed-forward neural network (FFNN) architecture used for the regression task. The input layer *L*_0_ consists of *p* = 55 neurons corresponding to the PL components for different control parameters β*_k_*. The hidden layers, with dimensions [*L*_1_, *L*_2_, *L*_3_,···], each employ the ReLU(·) activation function, while the output layer *L*_out_ contains three neurons corresponding to the predicted parameters Q .

The networks were implemented in PyTorch^79^ to perform supervised learning for regression tasks, exploring ten different architectures from 1 to 10 hidden layers. Training was carried out by minimizing a mean-squared-error (MSE) cost function between the true and predicted values of Q using the PL data set as input. Given the large amount of training data, the loss function was computed by dividing the data set into mini-batches. Consequently, the total loss is expressed as

12 $$C\left(Q,\mathrm{Q̃}\right)=\frac{1}{N}\overset{N/\mathrm{Bs}}{\underset{k=1}{\sum }}\underset{i\in M_{k}}{\sum }\left|\right|Q_{i}-{\mathrm{Q̃}}_{i}|{|}^{2}$$

where $Q_{i}\left({\mathrm{Q̃}}_{i}\right)$ denotes the target (predicted) values, and *M*_*k*_ is the *k*-th mini-batch with size Bs = |*M*_*k*_|. The training and validation sets contained $\frac{3}{5}N$ and $\frac{1}{5}N$ samples, respectively, where *N* is the total number of data points, while the remaining data were used as a test set to assess the network’s predictive performance and generalization ability. Training was performed for 500 epochs, with batch sizes (Bs) ranging from 32 to 256 and learning rates between 10^–1^ and 10^–6^, ensuring proper convergence of the parameters and stability of the training process.

For the regression task, we used a total of *p* = 55 predictors corresponding to the PL values at different β settings as input features to the network. The output layer was defined as a three-component vector representing the target parameters Q , with no activation function applied. The number of hidden layers [*L*_1_, *L*_2_,···, *L*_*h*_] was varied from *h* = 1 to *h* = 11 to evaluate the model performance and determine the optimal architecture that minimized the validation loss. Each hidden layer employed the ReLU activation function, defined as ReLU­(*x*) = max­{*x*, 0}, where the operation is applied element-wise over the vector components. The network was trained to determine the optimal weights and biases θ = {*W*_*i*_, *b*_*i*_} that minimize the cost function given in eq 12. For this task, the Adam optimizer^80^ was used, which evaluates the *n*-th gradient of the cost function *g* ^(*n*)^ = ∇_θ_ *C*(θ^(*n*)^) and, after random initialization, iteratively calculates the parameters θ^(*n*)^ until the stopping criterion is met. This approach allows for efficient and robust optimization of the network parameters, ensuring fast and stable convergence in a few epochs.

## Results and Discussion

As a first step, a Monte Carlo sweep was performed over the entire parameter space D to use these values as initial conditions for the optimizer, testing a total of 700 different values in order to analyze the parameters’ trending, convergence, and statistical errors.

In the first scenario, where all parameters were included, different values of the hyperparameter λ were tested in order to analyze the convergence behavior as a function of this regularization strength. For large values of λ ≫ 1, the optimization strongly enforced the constraint *E*_2_ → 2ω_0_, leading to a pronounced convergence toward this value but at the expense of a higher overall mean squared error (MSE). Conversely, for smaller values of λ = 10^–2^ and λ = 10^–3^, a more balanced behavior was achieved, in which the Ridge-like penalization^81,82^ applied to the parameter *E*_2_ remained significant while the total MSE was slightly affected. After collecting the final optimized parameters from all iterations, a histogram of the resulting values was constructed to visualize the distribution and identify emerging trends in the predicted parameters, as shown in Figure 7. This analysis allowed us to assess the stability of the optimization outcomes and the sensitivity of the extracted parameters with respect to variations in the regularization strength λ.

**Figure 7:**
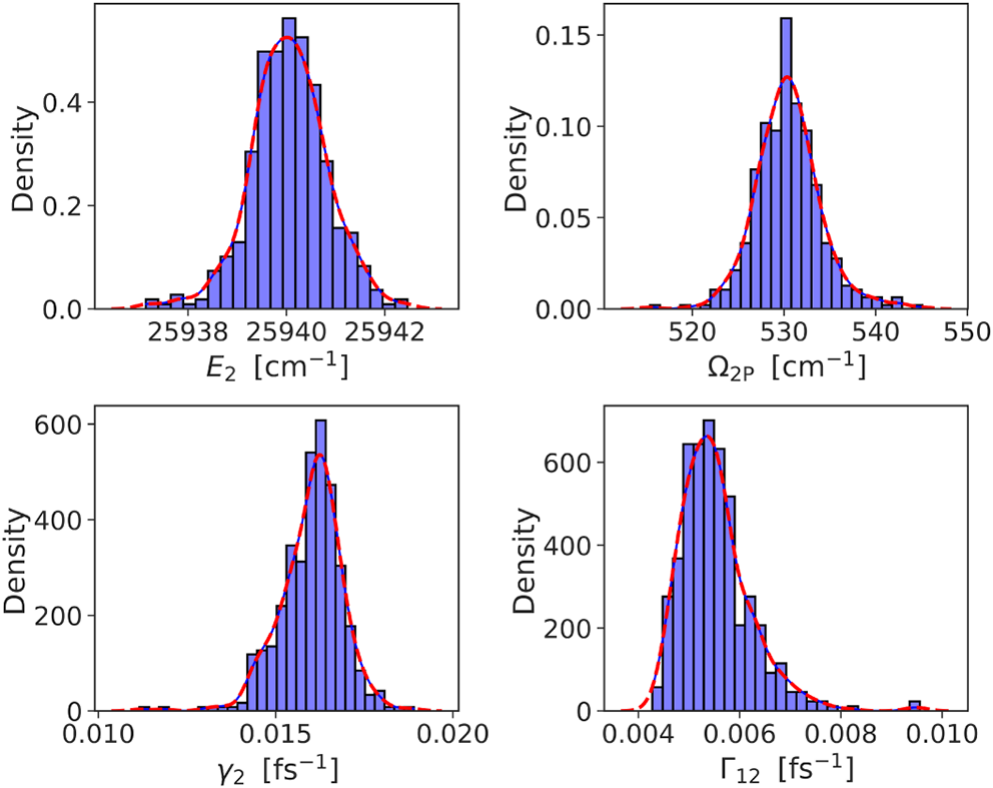
Histogram showing the distribution of the optimal parameter values $Q^{*}$ obtained for all iterations of the optimization algorithm using λ = 10^–2^, maxiter = 500, and ftol = 10^–4^.

From these histograms, the mean values obtained across all iterations were computed as the final results for each parameter. For the energy, a mean value of *E̅*_2_ = 25 940.04 cm^–1^ was obtained, together with a two-photon Rabi frequency of Ω̅_2P_ = 530.45 cm^–1^, a dephasing rate of *γ̅*_2_ = 0.016005 fs^–1^ and a decay rate of Γ̅_12_ = 0.0055336 fs^–1^. In contrast, for the second scenario, where λ = 0 and the energy was fixed at *E*_2_ = 25 940 cm^–1^, the corresponding mean values were Ω̅_2P_ = 533.04 cm^–1^, *γ̅*_2_ = 0.016495 fs^–1^, and Γ̅_12_ = 0.0050602 fs^–1^. These results confirm the numerical consistency between both scenarios, with only minor deviations attributable to the imposed regularization constraint on *E*_2_, which effectively reduces the parameter space from four to three variables.

For the neural network design, an initial sweep was performed over the possible architectures of the hidden layers to determine the optimal configuration that avoids overfitting in the training data while still capturing all relevant patterns and features of the input data to accurately predict the parameters. Figure 8 shows the Bias–Variance trade-off curve^81^ constructed using the validation set to compute the bias, variance, and total MSE loss. For all training runs and for performing bagging across architectures, 300 epochs were used with a learning rate of δ = 10^–3^ and a batch size of Bs = 256.

**Figure 8:**
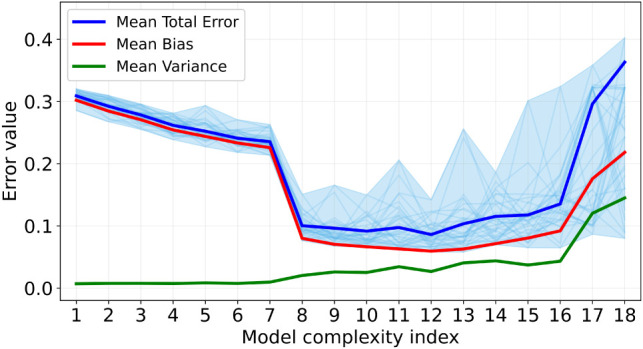
Bias–variance trade-off curve showing the mean total error, mean bias squared, and variance as a function of the model complexity of the neural network. The complexity indices are listed in the Supporting Information, Appendix A - Table 1. The semitransparent blue curves correspond to the ten new models generated through bagging,^83^ while the solid blue curve represents their average.

From the figure, it can be observed that the best trade-off point is achieved at index 12, which corresponds to an optimal architecture consisting of three hidden layers of sizes [128, 64, 32] (where the numbers refer to the number of nodes in each hidden layer). This architecture was then used to perform hyperparameter tuning on the remaining training parameters to achieve fast and stable convergence within the epochs. This hyperparameter sweep was carried out considering a fixed number of 200 epochs, batch sizes of Bs = {64, 128, 256, 512}, and learning rates of δ = {10^–4^, 10^–3^, 10^–2^, 10^–1^, 1}, while also including and excluding dropout in the activation functions of the hidden layers. The lowest validation loss was achieved with a batch size of Bs = 64, a learning rate of δ = 10^–4^, and a dropout rate of 0.0 (no dropout); see Supporting Information.

After being trained, the neural network was evaluated on the test set. For the three output parameter values, the performance metrics computed were root-mean-square error (RMSE), mean absolute error (MAE), and the *R* ^2^ coefficient.^84^ Since three different scaling and normalization methods were applied to the data, these metrics were evaluated separately for each case. The results of the corresponding performance metrics are summarized in Table 1.

**Table 1:** Performance Metrics of the Neural Network for Three Different Scaling Methods, Computed on the Test Dataset

Method	Target	RMSE	MAE	*R* ^2^
Standard Scaling	Ω_2P_	0.0283	0.0222	0.9992
	γ_2_	0.0346	0.0228	0.9989
	Γ_12_	0.1549	0.0850	0.9754
Robust Scaling	Ω_2P_	0.0318	0.0233	0.9990
	γ_2_	0.0365	0.0269	0.9987
	Γ_12_	0.1700	0.0947	0.9703
Robust Scaling + WP	Ω_2P_	0.0720	0.0340	0.9949
	γ_2_	0.0997	0.0387	0.9903
	Γ_12_	0.2879	0.1277	0.9182

After evaluating the average performance metrics of the neural network on the entire test data set, we proceeded to visualize the predictive accuracy of the model. Figure 9 shows the predicted values in comparison with the true (target) values Q . In this context, the *true values* refer to the ground-truth targets generated within the data set, while the *predicted values* correspond to the outputs produced by the FFNN. This comparison illustrates the overall agreement between the model outputs and the reference data as well as the deviations from the ideal perfect fit. The trend lines of the scattered points indicate the general behavior of the predictions over larger value ranges.

**Figure 9:**
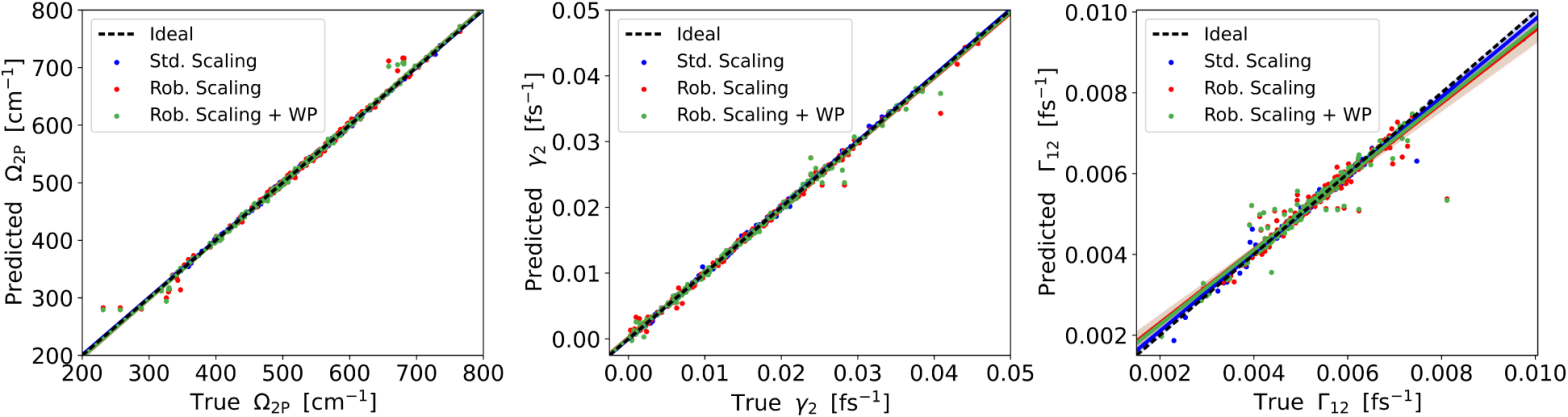
Predicted versus true values for the three target parameters $Q=\left[\Omega_{2P}\gamma_{2}\Gamma_{12}\right]$ using the three different scaling methods calculated over the test data set, highlighting deviations from the perfect one-to-one fit and including trend lines that illustrate the overall behavior and generalization of the predictions.

Finally, after training and validating the optimal neural network architecture and quantifying its performance on a test set containing samples unseen by the model, we proceeded to evaluate its predictive capability on the real experimental PL data of the MeLPPP molecule as reported in ref 52. This step provided a direct connection between the numerical model and the experimental observations, allowing us to assess the ability of the trained model to generalize beyond the simulated PL data set. Since three neural networks were independently trained using different data scaling and normalization strategies, the predictions of the physical parameters Q were evaluated separately for each of them. This comparison enabled us to identify how the input preprocessing affects the accuracy and robustness of the model when inferring the system parameters from experimental data. The results of this evaluation, together with the comparison between the optimization-based methods and the neural network predictions, are summarized in Table 2. In general terms, both algorithms performed well and successfully predicted the physical parameters associated with the molecular system, its environment, and the system–field coupling. However, depending on the specific target parameter, some methods outperformed others, as shown in Tables 1 and 2. Additionally, the uncertainty associated with these inferences on the real experimental data was quantified through statistical analysis and the use of Deep Ensembles,^85^ yielding mean predicted values and their respective uncertainties, as shown in Table 3. Further details on the application of these methods can be found in the Supporting Information, Appendix A.

**Table 2:** Comparison between Parameters Predicted by the Methods in This Work and the Parameters Values Obtained Using the QDI Protocol by Wilma *et al*.^52^ ^a^

	This work				Wilma et al.^52^
Target	Optimization Algorithm	NN with Std. Scaler	NN with Rob. Scaler	Rob. Scaler + WP	QDI
Ω_2P_	530.45 cm^–1^	531.78 cm^–1^	529.23 cm^–1^	525.27 cm^–1^	531 cm^–1^
γ_2_	0.016005 fs^–1^	0.014368 fs^–1^	0.015966 fs^–1^	0.016016 fs^–1^	1/61 fs^–1^
Γ_12_	0.0055336 fs^–1^	0.0026379 fs^–1^	0.0053537 fs^–1^	0.0049061 fs^–1^	1/190 fs^–1^

a Results are shown for both optimization-based and neural etwork approaches with different data pre-processing strategies.

**Table 3:** Results for the Uncertainty Quantification of the Optimization Algorithm and the Neural Network Models Using Deep Ensembles,^85^ Based on the Mean and Standard Deviation of the Ensemble Predictions

Method	Target	Mean (μ)	Std dev (σ)	Final report (μ ± σ)
Optimization Algorithm	Ω_2P_	530.45 cm^–1^	3.55 cm^–1^	530.45 ± 3.55 cm^–1^
	γ_2_	0.016005 fs^–1^	0.000884 fs^–1^	0.016005 ± 0.000883 fs^–1^
	Γ_12_	0.005533 fs^–1^	0.000696 fs^–1^	0.005533 ± 0.000696 fs^–1^
NN with Std. Scaler	Ω_2P_	542.38 cm^–1^	10.78 cm^–1^	542.38 ± 10.78 cm^–1^
	γ_2_	0.015691 fs^–1^	0.001125 fs^–1^	0.015691 ± 0.001125 fs^–1^
	Γ_12_	0.002378 fs^–1^	0.000247 fs^–1^	0.002378 ± 0.000247 fs^–1^
NN with Rob. Scaler	Ω_2P_	525.89 cm^–1^	2.44 cm^–1^	525.89 ± 2.44 cm^–1^
	γ_2_	0.016385 fs^–1^	0.000305 fs^–1^	0.016385 ± 0.000305 fs^–1^
	Γ_12_	0.004508 fs^–1^	0.000171 fs^–1^	0.004508 ± 0.000171 fs^–1^

Regarding the first approach based on the optimization algorithm, despite its remarkable sensitivity to the choice of initial parameters during the minimization process, it was observed that when sampling across a wide range of initial conditions, the resulting estimates follow a consistent trend that agrees with the typical values reported in the literature.^52,53^ Consequently, this method presents the drawback that the physical parameters Q cannot be reliably extracted from experimental PL data in a single optimization run. Instead, a broad sampling of initial conditions is required to reveal clear and stable parameter trends. Additionally, for the algorithm to operate properly, it is crucial to define appropriate physical constraints to ensure robust convergence. In turn, this requires prior knowledge or qualitative insights into the molecular system under coherent control, such as its relevant electronic states and lifetimes or the energetic scales of the driving and dissipative processes, including temperature and solvent effects.^6,14,14,41,42^ These considerations are essential for defining meaningful initial conditions and parameter boundaries, leading to results that have a reasonable and physically interpretable meaning.

On the other hand, the neural network approach eliminates the need to perform multiple optimization runs to obtain reliable parameter estimates on real experimental data. Once properly trained and validated, the NN can evaluate any systematic set of experimental PL data and immediately extract the corresponding physical parameters. However, this advantage comes with the requirement of an extensive training and validation phase involving the exploration of different network architectures, feature-scaling methods, and hyperparameter configurations. Moreover, the performance and generalization capability of the NN are inherently limited by the quality and representativeness of the training data,^86^ which, in this case, are generated from a photoluminescence model assuming collective emission as the sum of individual single-molecule contributions.^34^ Nevertheless, when the simulated PL data are produced under well-controlled experimental conditions (e.g., fixed emission intensities, pulse duration, temperature, and molecular parameters), the trained model can successfully predict physically meaningful values that closely agree with those reported experimentally.^52^ In addition, evaluation metrics obtained for the test set and real PL data demonstrate that the trained model achieves a strong predictive performance. Additionally, it was observed that the feature-scaling method applied to the input data can slightly influence the training dynamics and the resulting predicted targets. This highlights the importance of selecting an appropriate normalization scheme during preprocessing.

## Conclusions

In this work, we developed and validated robust algorithms that extract physically meaningful parameters associated with environmental interactions and driving couplings from experimentally accessible photoluminescence observables. We use the MeLPPP single molecule as a sample system. Our approach combines a description of the molecule’s ultrafast dynamics using a Lindblad master equation framework with a PL forward model mapping pulse, molecular, and environmental control parameters to the normalized PL intensity. This PL model formed the basis for developing the two complementary algorithms for parameter optimization and extraction using real experimental data.

Despite the well-known sensitivity of the inverse problem to initial conditions and the existence of multiple numerical solutions, the first approach, a direct inverse optimization algorithm, can reliably recover parameter estimates when they are characterized statistically. By running multiple independent optimizations, we obtained distributions that, while variable across individual runs, systematically converged to mean values consistent with the previously reported parameters. We strengthened the cost function with additional terms that provide physical insight, creating a more robust model, and thoroughly tested the statistical behavior of the predictions, comparing its performance with that of similar algorithms.

The second approach involves training a feed-forward neural network to predict dissipative and coupling parameters from the PL data. During training, the network learns to identify correlations between the input PL traces and the dissipation and molecular parameters Q . It adjusts its internal weights to construct a mapping between diverse PL signals and underlying environmental parameters. This mapping enables the network to generalize well to unseen data and produce physically reasonable parameter estimates when applied to real PL traces. Since statistical characterization and validation are embedded in the training stage, inference on new molecular experimental data requires only one forward pass through the network, enabling fast, reliable parameter estimation.

Table 2 summarizes the main findings and shows that both methods predict parameter values consistent with earlier reports using PL under spectral phase modulation. The two approaches offer complementary advantages. The inverse optimization method is straightforward and interpretable, although it requires repeated runs to assess uncertainty. In contrast, the neural network approach provides rapid single-shot inference, albeit with a more involved preprocessing, training, and architecture selection phase.

Overall, this work provides robust, validated tools for translating PL observables into quantitative physical parameters. This makes them practical for future coherent control experiments on single quantum objects and real-time experimental workflows. Future work will focus on extending the framework to broader classes of molecules and artificial atoms, incorporating experimental noise models during training, and integrating the methods into adaptive measurement schemes. Additionally, considering physical models beyond the standard Lindblad master equation could significantly expand the parameter space associated with the environment. Examples of such models include structured baths with temperature-dependent spectral densities, which offer a general, non-Markovian, broadband description of the dissipative processes.

## Supplementary Material



## Notes

In-depth analysis of the implementation, training, and hyperparameter tuning of the neural network method; additionally, we provide plots obtained during the statistical analysis of the optimization algorithm to generate histograms of the predictions (PDF)
