# Supplementary material for: Deep Learning Parameter Estimation and Quantum Control of Single Molecules
Source: ACS Phys Chem Au. 2026 Jun 12;6(4):858–68. doi: 10.1021/acsphyschemau.6c00048 (PMC13397457; doi:10.1021/acsphyschemau.6c00048)
Supplement: Supplementary file 1 [file pg6c00048_si_001.pdf]

Supplementary Information: Deep learning parameter estimation and quantum control of single molecules

Juan M. Scarpetta[1,2], Omar Calderón-Losada[1], Morten Hjorth-Jensen[2], and John H. Reina[1,2*]

[1]Department of Physics and Centre for Bioinformatics and Photonics—CIBioFi, Universidad del Valle, Cali 760032, Colombia

[2]Department of Physics and Center for Computing in Science Education, University of Oslo, Oslo N-0316, Norway

*Email: john.reina@correounivalle.edu.co

Neural network training

In the Methods Section of main text, we described the training strategy employed for the neural network. As a preliminary step, exploratory tests were conducted to identify the optimal hyperparameters for the subsequent training and validation procedures. These tests focused on determining an appropriate network architecture with enough hidden layers to provide sufficient capacity to prevent underfitting and adequately capture the relevant structures relating the input PL data to the output parameters $\mathcal{Q}$, while simultaneously avoiding excessive complexity that could lead to overfitting. To perform this analysis, we used a learning rate of $\delta = 10^{-3}$, a batch size of 256 and no dropout, and we trained each model for up to 100 epochs with early stopping using a patience of 30 epochs. This procedure was used to train and record the validation error for each architecture considered, spanning from one to eleven hidden layers. The validation MSE was further decomposed into its bias-squared and variance contributions. In order to obtain robust estimates, the total validation error for each architecture was bagged over 10 independent validation curves. The results are summarized in Figure 8 of main text and the definitions of the corresponding indices are provided in Table 1.

Once the optimal network hidden layers architecture of [128,64,32] was determined, the remaining hyperparameters were optimized, keeping this architecture fixed. We set fixed training conditions of 200 epochs and a patience of 30 epochs and performed a grid search over batch sizes $\text{Bs} = \{64, 128, 256, 512\}$ and learning rates $\delta = \{1, 10^{-1}, 10^{-2}, 10^{-3}, 10^{-4}\}$. The resulting training and validation errors are presented in Figure 1.

ID	Model Sizes
1	[8]
2	[12]
3	[16]
4	[20]
5	[24]
6	[28]
7	[32]
8	[64, 32]
9	[76, 48]
10	[88, 60]
11	[128, 64]
12	[128, 64, 32]
13	[256, 128, 64, 32]
14	[512, 256, 128, 64, 32, 16]
15	[1024, 512, 256, 128, 64, 32, 16]
16	[2048, 1024, 512, 256, 128, 64, 32, 16]
17	[4096, 2048, 1024, 512, 256, 128, 64, 32, 16, 4]
18	[8192, 4096, 2048, 1024, 512, 256, 128, 64, 32, 16, 4]

Table 1: Representation of the model complexity indices **ID** shown in Figure 8 of main text, mapping them to the corresponding neural network hidden layer architectures (model complexity). The numbers in the parentheses refer to number of nodes in each layer. The deepest network has eleven hidden layers and is given by **ID** 18.

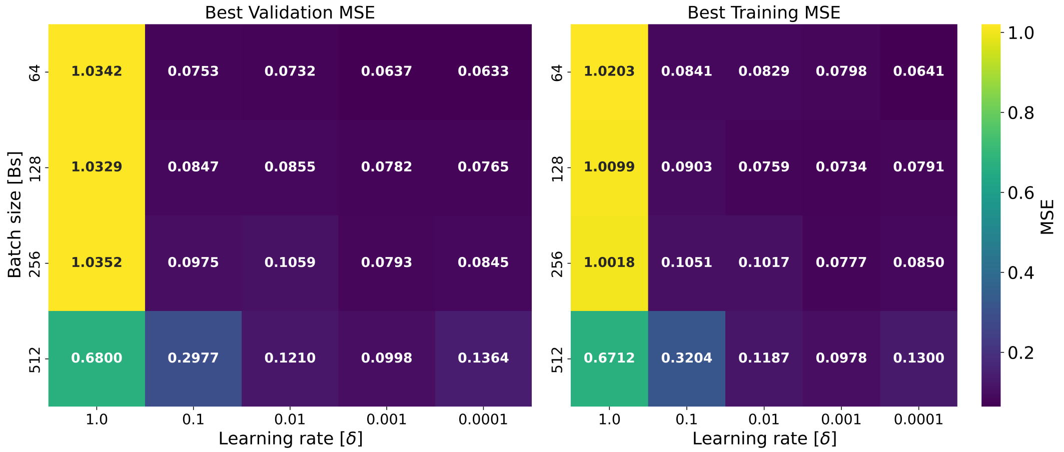

Figure 1: Heatmaps of the best MSE achieved across the hyperparameter grid. The left panel shows the best validation MSE, and the right panel shows the best training MSE. It is seen that the minimum MSE within 200 epochs is achieved for $\delta = 10^{-4}$ and $\mathrm{Bs} = 64$.

From Figure 1, it is clear that a simultaneous minimum in both the training and validation MSE occurs for the configuration with a learning rate $\delta = 10^{-4}$ and a batch size $\mathrm{Bs} = 64$. These values were therefore adopted as the optimal hyperparameters for training the network. In Figure 2, these profiles are further illustrated by showing their evolution over the course of the training epochs.

After identifying these hyperparameters, we analyzed the effect of including dropout in the hidden layers

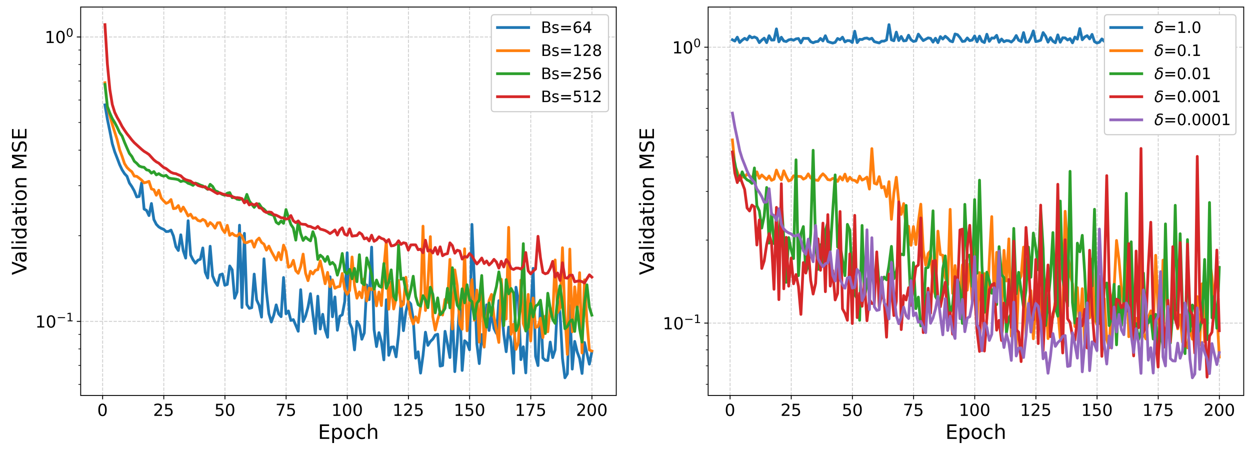

Figure 2: Validation MSE as a function of training epochs. The left panel shows curves for a fixed learning rate with varying batch sizes, while the right panel shows curves for a fixed batch size with varying learning rates. It is shown that a batch size $\mathrm{Bs} = 64$ outperforms larger batch sizes, while learning rates on the order of 10^{-3} or lower exhibit similar convergence behavior.

to determine the optimal dropout rate for training. The results are shown in Figure 3 as a function of different dropout probabilities p.

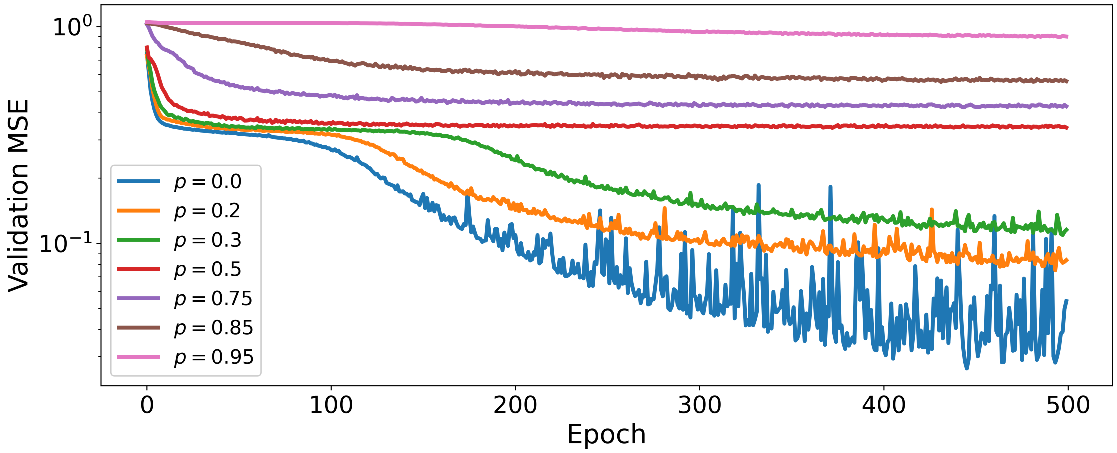

Figure 3: Test training for different dropout rates p, using a learning rate of $\delta = 10^{-3}$, a batch size of 64 and a total of 500 fixed epochs without a scheduler. It is clear that the fastest convergence and lowest validation error are obtained for $p = 0$, that is, with no dropout.

Deep Ensembles

To quantify uncertainty in neural network predictions, we use the *Deep Ensembles* approach. An ensemble of K networks is trained using bootstrapped datasets and independent random initializations. Each model yields a set of trained weights $\theta^{(1)}, \theta^{(2)}, \ldots, \theta^{(K)}$, thereby defining the predictive models $f_\theta(x)$. Each model then produces a prediction for the experimental data x^*, with output parameters given by $\hat{y}^{(k)} = f\left(x^*; \theta^{(k)}\right)$ for $k = 1, 2, \ldots, K$. From the ensemble predictions $\hat{y}^{(1)}, \hat{y}^{(2)}, \ldots, \hat{y}^{(K)}$, we compute the mean μ and the covariance matrix $\mathbf{\Sigma}$ as

$$\mu = \frac{1}{K}\sum_{k=1}^{K}\hat{y}^{(k)} \qquad \boldsymbol{\Sigma} = \frac{1}{K-1}\sum_{k=1}^{K}\left(\hat{y}^{(k)} - \mu\right)\left(\hat{y}^{(k)} - \mu\right)^{T}. \tag{1}$$

The uncertainty associated with the three output parameters is then quantified through the standard deviations $\sigma_j = \sqrt{\boldsymbol{\Sigma}_{jj}}$ for $j = 1, 2, 3$.

Convergence with K

We ran the simulations using an ensemble of $K = 50$ networks. For each of these, we computed the inferences over the experimental data, calculating the mean value and covariance matrix over the ensemble. To check for convergence, we calculated the error between the mean values and the ensemble mean to determine the optimal value of K. Similarly, we computed the error of the covariance matrix with respect to the ensemble using the Frobenius norm. The results are shown in Figure 4.

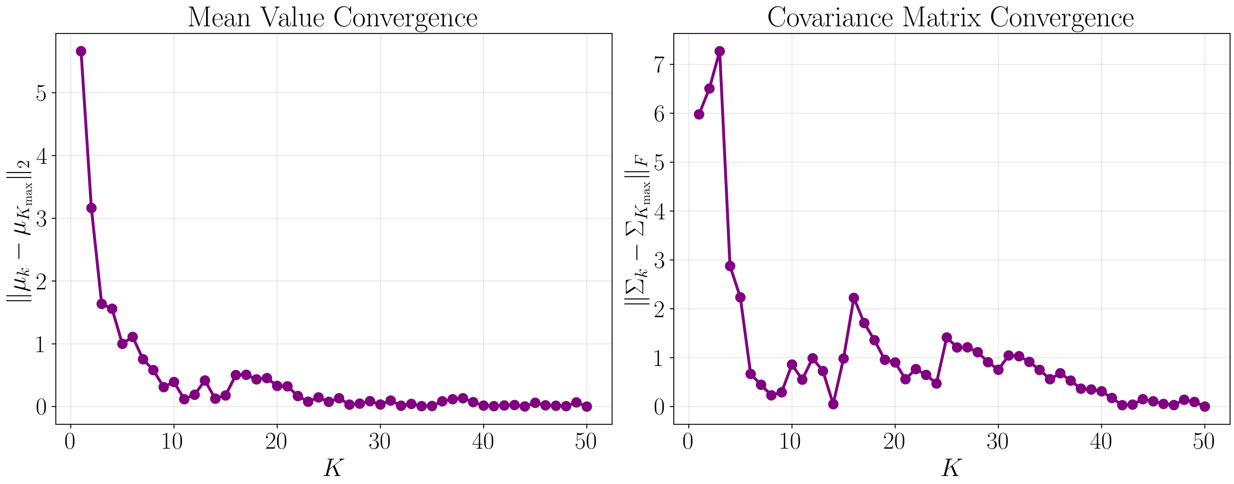

Figure 4: Convergence of the ensemble mean (left) and the covariance matrix (right) as a function of the model index k, computed over an ensemble of $K = 50$ networks.

We also computed the convergence of the standard deviation as a function of the number of ensembles K. The results are shown in Figure 5.

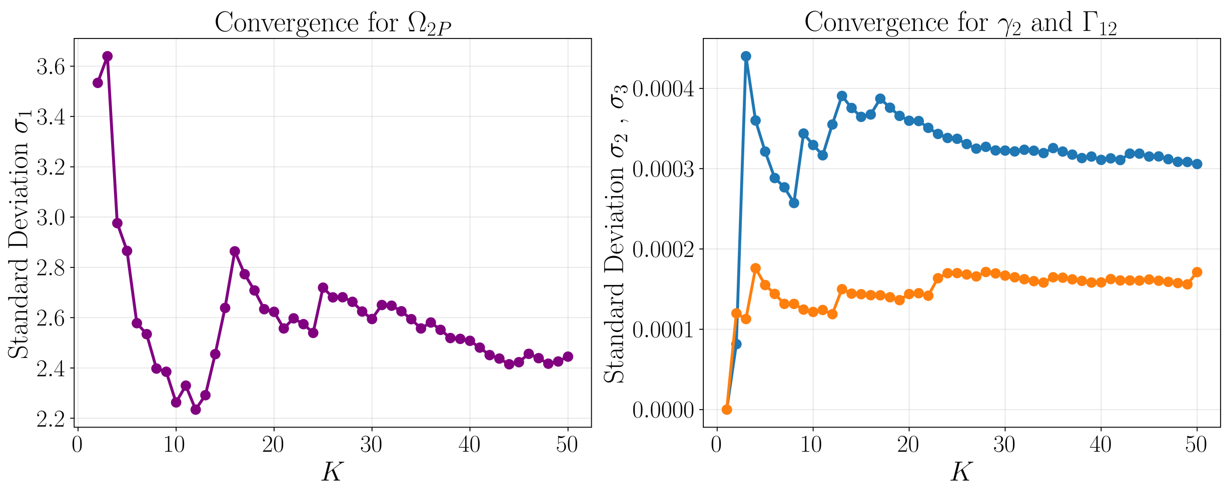

Figure 5: Convergence of the standard deviation with K for the three output parameters: the two-photon Rabi frequency (left), and the dephasing and relaxation rates (right).

Finally, we calculated the total predictions and metrics for the ensemble of $K = 50$ networks. We retrieve the mean value, the variance and the standard deviation of each output parameter. The results are presented in Tables 2 and 3.

Parameter	Mean value	Standard deviation (σ)	Final report ($\mu \pm \sigma$)
Ω_{2P}	542.38 cm^{-1}	10.78 cm^{-1}	542.38 ± 10.78 cm^{-1}
γ_2	0.015691 fs^{-1}	0.001125 fs^{-1}	0.015691 ± 0.001125 fs^{-1}
Γ_{12}	0.002378 fs^{-1}	0.000247 fs^{-1}	0.002378 ± 0.000247 fs^{-1}

Table 2: Statistical results of parameter extraction using **standard scaling.**

Parameter	Mean value	Standard deviation (σ)	Final report ($\mu \pm \sigma$)
Ω_{2P}	525.89 cm^{-1}	2.44 cm^{-1}	525.89 ± 2.44 cm^{-1}
γ_2	0.016385 fs^{-1}	0.000305 fs^{-1}	0.016385 ± 0.000305 fs^{-1}
Γ_{12}	0.004508 fs^{-1}	0.000171 fs^{-1}	0.004508 ± 0.000171 fs^{-1}

Table 3: Statistical results of parameter extraction using **robust scaling.**

Optimization Algorithm

For the optimization algorithm described in Methods Section, once all iterations across all initial conditions were completed, the histograms shown in Figure 7 of main text were obtained. Figures 6 and 7 provide additional details illustrating how these histograms are generated as the iterations progress and how the error is gradually minimized toward its final value.

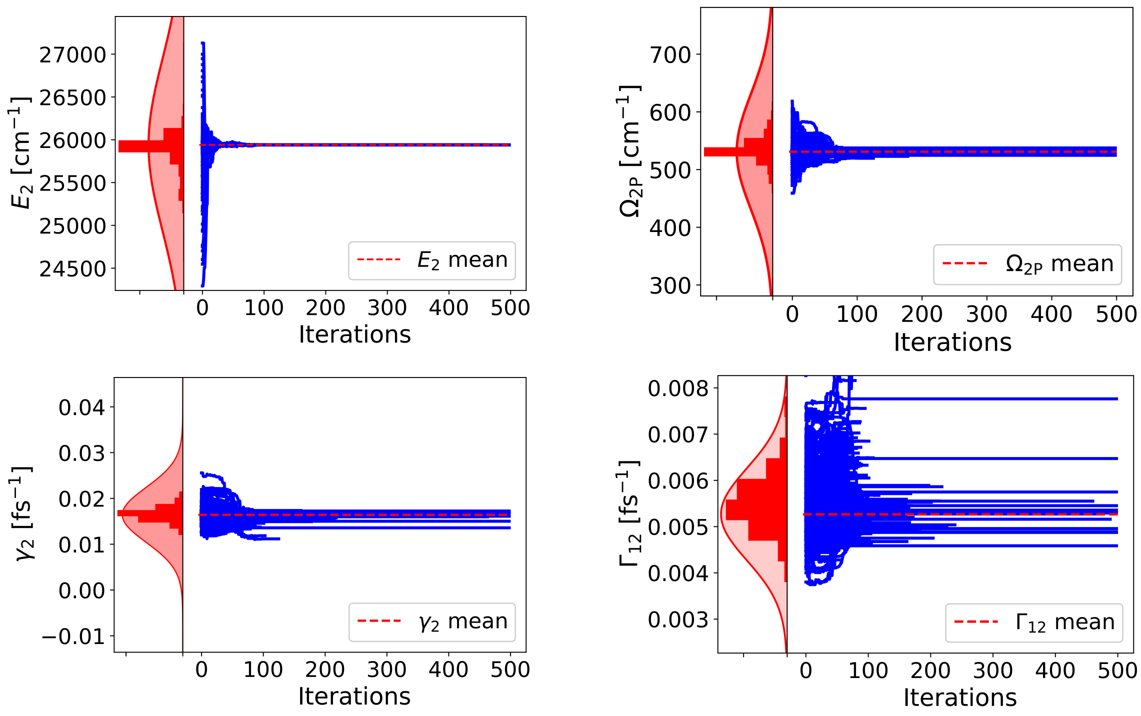

Figure 6: Evolution of the target parameters across iterations of the optimization algorithm using $\lambda = 10^{-2}$ in the scenario with four free parameters.

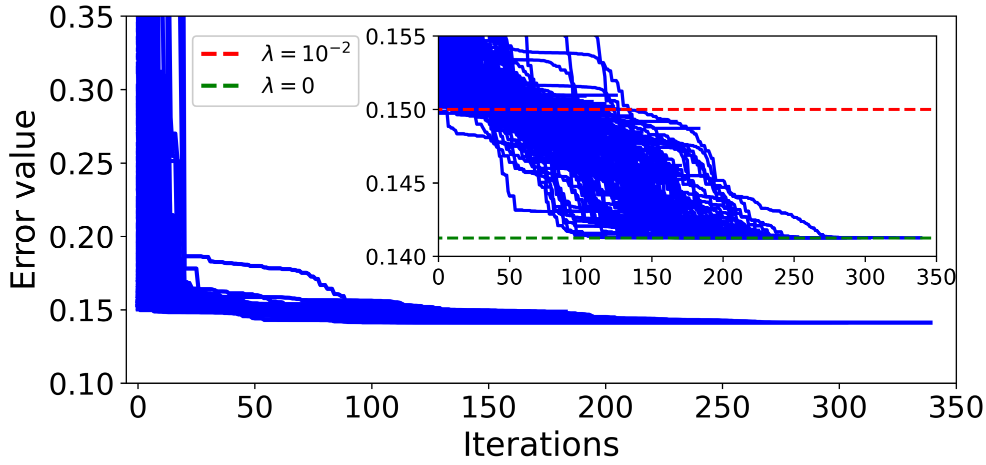

Figure 7: Evolution of the mean error between the theoretical PL prediction and the experimental data as a function of the iteration number. After approximately 100 iterations, the error decreases rapidly and reaches convergence. The dashed red and green lines highlight the final error values for the cases $\lambda = 10^{-2}$ and $\lambda = 0$, respectively.

This method involves running the algorithm several times to obtain the histograms presented in the main article. The mean values of these distributions are then used to make the final predictions for the experimental data. Here, we extend the statistical analysis to quantify the uncertainty of the algorithm's outputs by calculating the standard deviation of the predicted values. The results are summarized in Table 4.

Parameter	Mean value (μ)	Standard deviation (σ)	Final report ($\mu \pm \sigma$)
$\Omega_{2\mathrm{P}}$	530.45 cm^{-1}	3.55 cm^{-1}	530.45 ± 3.55 cm^{-1}
γ_2	0.016005 fs^{-1}	0.000884 fs^{-1}	0.016005 ± 0.000883 fs^{-1}
Γ_{12}	0.005533 fs^{-1}	0.000696 fs^{-1}	0.005533 ± 0.000696 fs^{-1}

Table 4: Results of the statistical analysis of the Optimization Algorithm.

Correlation Matrices

We quantify the correlations between variables across the ensemble predictions by computing the correlation matrix from the corresponding variances and standard deviations, as shown in Figure 8.

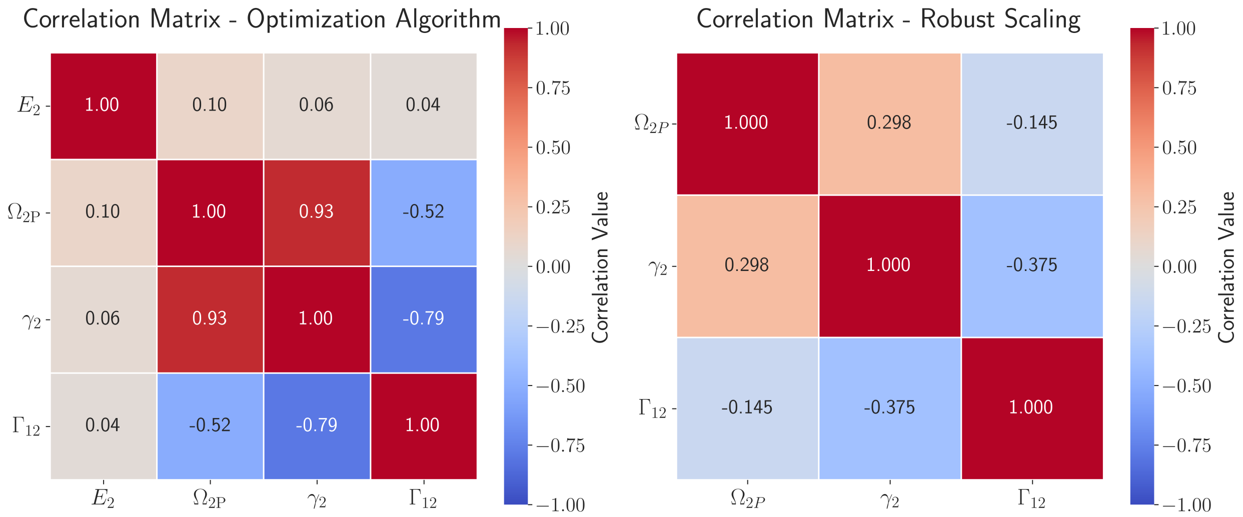

Figure 8: Correlation matrices for the models.

These results allow us to infer the correlations among all the model parameters. Firstly, we observe that the energy E_2 is only weakly correlated with the other parameters, as this quantity is not directly associated with environmental dissipation. Conversely, the strongest correlation in both models is observed between the parameters γ_2 and Γ_{12}. This suggests the existence of multiple feasible solutions to the optimization problem, while the predictions nevertheless exhibit a statistical tendency toward stable values, as reflected in the histograms and the Deep Ensembles analysis.
